# Maternal Left Ventricular Diastolic and Systolic Function during Normal Pregnancy in Saint Bernard Dogs

**DOI:** 10.3390/vetsci8120306

**Published:** 2021-12-04

**Authors:** Luís Lobo, Joana Esteves-Guimarães, Pedro Oliveira, Luís Salazar, André Pereira, Ana Patrícia Fontes-Sousa

**Affiliations:** 1 Hospital Veterinário do Porto, 4250-475 Porto, Portugal; luis.lobo@onevetgroup.pt (L.L.); luismps@msn.com (L.S.); 2 Faculdade de Medicina Veterinária, Universidade Lusófona de Humanidades e Tecnologias, Campo Grande, 376, 1749-024 Lisboa, Portugal; 3 Centro de Estudos de Ciência Animal (CECA), Instituto de Ciências, Tecnologias e Agroambiente da Universidade do Porto (ICETA), Rua D. Manuel II, Apartado 55142, 4051-401 Porto, Portugal; 4 Clínica Veterinária de Ovar, 3880-251 Ovar, Portugal; Joana.eg@gmail.com; 5 Davies Veterinary Specialists, Manor Farm Business Park, Hitchin SG5 3HR, UK; pedro.oliveira@vetspecialists.co.uk; 6 Centro Hospitalar Veterinário, 4100-320 Porto, Portugal; andrepereira@chv.pt; 7 Centro de Investigação Farmacológica e Inovação Medicamentosa (MedInUP), Departamento de Imuno-Fisiologia e Farmacologia, ICBAS—Instituto de Ciências Biomédicas Abel Salazar, Universidade do Porto, Rua Jorge de Viterbo Ferreira 228, 4050-313 Porto, Portugal

**Keywords:** dog, maternal cardiac function, doppler echocardiography, tissue doppler imaging

## Abstract

This study aimed to evaluate maternal left ventricular (LV) systo-diastolic function using conventional and TDI echocardiography and included 10 healthy Saint-Bernard pregnant bitches. M-mode, peak transmitral flow velocities during early diastole (E) and atrial contraction (A), aortic and pulmonic flow, myocardial performance index (MPI), TDI studies (peak myocardial velocities during early diastole (E’), atrial contraction (A’) and peak systole (S’)), and blood pressure were measured at 21 to 28 (T1), 40 (T2) and 60 (T3) days of gestation and four to eight weeks postpartum (T4). Cardiac output and heart rate were 20% and 9% higher at T3, respectively, compared to T4 (*p* < 0.01). Lateral S’ was 36% higher at T3 than at T1 (*p* < 0.05). Changes in diastolic function were demonstrated by 10% lower E wave and 15% A wave at T1, compared to T4 (*p* < 0.05). E’ and A’ were 23% and 42% higher at T3 compared to T4 (*p* < 0.01). Both lateral E/E’ and E’/A’ were 6% and 19% lower at T3 compared to T1 (*p* < 0.01 and *p* < 0.05, respectively). At T3, MPI was 51% and 34% lower when compared to T1 or T2 (*p* < 0.05). The echocardiographic evaluation of maternal cardiac function is important, as structural, and functional changes occur throughout pregnancy.

## 1. Introduction

Appropriate development of the foetuses during pregnancy induces several hemodynamic alterations, with consequent cardiac changes. Echocardiographic and Doppler studies have been widely used to assess heart function during pregnancy in humans [1,2,3] and in dogs [4,5,6,7,8].

Pulsed wave Doppler transmitral inflow velocity may not truly reflect the left ventricle (LV) diastolic function during pregnancy since it is highly influenced by ventricular loading conditions, which are naturally altered by the pregnancy volume overload state [9]. Tissue Doppler imaging (TDI) is an echocardiography technique used to estimate myocardial velocities during contraction and relaxation, which measures high-intensity, low-velocity myocardial echoes; therefore, it directly evaluates the primary events of myocardium, being relatively independent of preload [9,10]. Significant changes in TDI parameters associated with no changes in conventional Doppler mode are reported [10], and TDI alterations are detected earlier, since longitudinal function may deteriorate prior to circumferential function [1]. TDI evaluation of cardiac dysfunction in veterinary medicine has been reported, highlighting the sensitivity of this technique in several cardiac diseases [11,12].

Myocardial performance index (MPI, also known as Tei Index) is a valuable tool in evaluating systo-diastolic function. This index correlates with the New York Heart Association (NYHA) class and some echocardiographic indices (e.g., ejection fraction and ventricular volumes), serving as a prognostic measure [13]. In addition, MPI is independent of ventricular geometry, preload, and heart rate [14,15].

The cardiac changes in human pregnancy have been widely studied. A primary fall in vascular tone in early pregnancy leads to a rapid decrease in preload and afterload with compensatory increase in heart rate and activation of the volume-restoring mechanisms, with resulting increase in cardiac output [16]. This compensatory volume overload induces an increase in left atrium size [3] and LV filling rates (E’ and A’) by the second trimester [9]. With advancing gestation age, the consequent stretching of myocardial fibers produces myocardial hypertrophy [17], with a decrease in LV compliance [18] that leads to a decrease in early diastolic filling rate (E’), with the left atrium becoming responsible for the ventricle filling rate (increase A’) by late pregnancy [9].

In the bitch, significant changes have been observed by late pregnancy, characterized by an increase in heart rate [4,19], stroke volume and cardiac output [4,7]. Volume overload and ventricular hypertrophy also occurred in late pregnancy [6,7]. Some studies documented an enhanced systolic performance in mid-late pregnancy [4,5,6,8], contrary to others [7], and some degree of diastolic impairment [4,6].

Understanding the physiological adaptations of the heart to the pregnancy is essential for monitoring maternal cardiac function and detecting early signs of cardiac failure in high-risk pregnancies [20] or obstetric complications due to cardiac maladaptation [6]. Additionally, the study of healthy dogs of a large breed could provide important information, as they are especially prone to developing cardiac diseases [21]. The aim of this study was to describe the adaptive cardiac changes during normal pregnancy in healthy Saint Bernard bitches, as well as providing normal ranges for several echocardiographic systolic and diastolic indices in these dogs, using conventional and TDI echocardiography.

## 2. Materials and Methods

### 2.1. Animals and Follow-Up

Ten healthy Saint-Bernard bitches, aged between 17 and 74 months (42.14 ± 21.5 months), with 59.67 ± 5.93 kg at first presentation, were included in the study. Pregnancy diagnosis was made by ultrasound (GE Vivid 3 PRO^®^, with a 3.5-MHz linear transducer), at the first visit, and the gestational age was calculated from the date of mating. The bitches were evaluated three times during pregnancy at early (days 21–28, T1), mid (day 40, T2) and late pregnancy (day 60, T3), as well as 4 to 8 weeks postpartum (PP). In each visit, the dogs were evaluated by clinical examination, obstetric ultrasound for monitoring foetal development [22], echocardiography and measurement of arterial pressures. Study participants were privately owned dogs. All owners were informed about the purpose of the study and gave their written consent.

### 2.2. Echocardiographic Evaluation

Conventional echocardiography (2D, M-mode and Doppler) and TDI were performed using a 3.5–7.5 MHz phased array transducer, GE Vivid 3 PRO^®^ echocardiograph, after a resting period of 15 min. Data were digitally stored for posterior measurements, according to the Echocardiography Committee of the Specialty of Cardiology (ACVIM) [23]. Acoustic gel was placed over the transducer and applied directly to the clipped skin. No sedation was required. To minimize variability, three consecutive measurements were recorded for each parameter by a single trained operator, as previously recommended [24].

For the right parasternal views, the bitches were placed in right lateral recumbency. From the right parasternal short-axis view, 2-dimensional (2D), guided M-mode tracings were made just below the mitral valve, at the level of the papillary muscles for measurements of the interventricular septum (IVS), LV internal dimension (LVID) and LV posterior wall (LVPW) in diastole (d) and systole (s). The right parasternal long-axis view with 2D-guided M-mode was used for the measurements of the E-point-to-septal separation interval (EPSS) in the plane of mitral valves.

The aortic (Ao) and left atrium (LA) diameters and the LA/Ao ratio were evaluated by the right parasternal short-axis view at the level of the aortic valve. The fractional shortening (FS) and the ejection fraction (EF) of LV were calculated according to the formulas: FS (%) = [(LVIDd − LVIDs) /LVIDd] × 100 and EF = [(LVIDd^3^ − LVIDs^3^)/LVIDd^3^] × 100 [25].

Pulmonary flow velocities were determined by using pulsed-wave Doppler from the right parasternal short axis view. Aortic and mitral flows were assessed with pulsed-wave Doppler from the left parasternal apical 5- and 4-chamber views, respectively. For the transmitral flow, the sample volume was placed just above the mitral valve leaflets during diastole, and the peak flow velocities during early diastole (E-wave) and during atrial contraction (A-wave) were measured.

Heart rate (HR) was calculated directly from the inter-beat intervals of the pulsed-wave aortic spectrogram. Stroke volume (SV) was derived by the formula SV = EDV − ESV, where EDV is the end diastolic volume calculated by EDV = [7 × (LVDd/10)^3^]/(2.4 + LVDd/10) and ESV is the end systolic volume, calculated by ESV = [7 × (LVDs/10)^3^]/(2.4 + LVDs/10). Cardiac output (CO) was calculated as the product of SV and HR, and the LV mass was calculated by the formula: 0.8 × [1.04 × (((ISVd/10 + LVDd/10 + LVPWd/10)^3^) − (LVDd/10)^3^)] + 0.6 [25].

Left ventricular myocardial performance index (MPI), also known as Tei Index, was calculated from pulsed-wave Doppler, according to the formula: (IVCT+IVRT)/ET, where IVCT, IVRT and ET represent, respectively, isovolumetric contraction time, isovolumetric relaxation time, and left ventricular ejection time [26]. Value for IVCT was measured from the end of mitral A wave to the onset of the left ventricular outflow tract, where value for IVRT was measured from the end of the left ventricular outflow tract to the onset of mitral E wave. ET was measured from the onset to the end of the left ventricular outflow tract pulsed-wave Doppler tracing.

Spectral TDI recordings were made from the apical four-chamber, left parasternal view. Ventricular myocardial velocities were assessed during both contraction and relaxation. The sample volume was placed at the lateral margin (lateral wall) and at the common septal margin (septal wall) of the mitral annulus, as previously described [11]. From the myocardial velocity patterns obtained, peak myocardial velocities during early diastole (E’), atrial contraction (A’), and peak systole in ejection phase (S’) were measured. The ratio of E’ to A’ and the ratio of transmitral E velocity to E’, an index of left ventricular filling pressure, were calculated. To minimize variability, three representative cardiac cycles were analysed, and a mean value was calculated for each measurement.

### 2.3. Blood Pressure Measurement

Blood pressures measurement was performed as previously described [27]. The bitches were placed in right lateral recumbency in a quiet room, after a resting period of 15 min, and an appropriate-size cuff was placed around the left forearm. A high-definition oscillometer device (Memodiagnostic^®^) was used to collect five consecutive measurements, after discarding the first one, from which the means for systolic (SAP; mmHg), diastolic (DAP; mmHg) and mean (MAP; mmHg) arterial pressures were calculated.

### 2.4. Statistical Methods

Data were graphed and analysed using the Prism 9.0 software (GraphPad Software, San Diego, CA, USA). Data were expressed as mean and standard deviation (SD). Comparisons between groups were analysed by one-way analysis of variance (ANOVA), that compared echocardiographic variables obtained at early, mid and late pregnancy as well as at postpartum. Post hoc evaluation was performed using the Tukey comparison test. *p* < 0.05 was considered statistically significant.

## 3. Results

Selected echocardiographic variables are listed in Table 1. The A wave (and respective E/A ratio) and EPSS were not possible to record in one and three dogs, respectively, because of technical difficulties. Repeated measures ANOVA disclosed a statistically significant effect of time on: body weight, arterial pressures (SAP, MAP and DAP), heart rate, cardiac output, LA/Ao ratio, mitral early diastolic E wave, mitral late diastolic A wave, pulmonic and aortic Doppler-derived flow, MPI, TDI lateral velocities (S’, E’, A’), lateral E’/A’ ratio, and lateral E/E’ ratio. There was no statistically significant effect of time on SV, LV dimensions (LVIDd, LVIDs, LVPWd, LVPWs, IVSd, IVSs), LV mass, FS, EF, mitral E/A ratio, TDI septal velocities (S’, E’ and A’), septal E’/A’ ratio, and septal E/E’ ratio.

Post hoc testing revealed several statistically significant differences. Body weight was significantly higher on late pregnancy (T3) than at T1, T2 or postpartum (*p* < 0.01). Blood pressure (SAP, MAP and DAP) was higher at T1, T2 or T3 than at postpartum (*p* < 0.01) and reached maximal values at T1 that progressively decreased until the end of pregnancy (T3).

Heart rate was 10% and 9% higher at T3 when compared to T1 or postpartum, respectively (*p* < 0.05), and the cardiac output was 20% higher at T3 than at postpartum (*p* = 0.02). The LA/Ao ratio was 7% higher at T2 than at T3 (*p* = 0.03). Transmitral early diastolic flow (E) was 9% lower at T1 than at T2 or T3 (*p* < 0.05). At T1, transmitral late diastolic flow (A) was 13%, 16% and 15% lower than at T2, T3 or postpartum, respectively (*p* < 0.05). Pulmonic flow was 26% and 21% greater at T3 than at T1 or T2, respectively (*p* < 0.05). Similarly, aortic flow was also 13% higher at T3 than at T1 (*p* < 0.01).

Even though ejection time did not change throughout pregnancy, both IVRT+IVCT and MPI decreased, with the latter being 56% and 34% lower at T3 when compared to T1 or T2 (*p* < 0.05).

**Figure 1:**
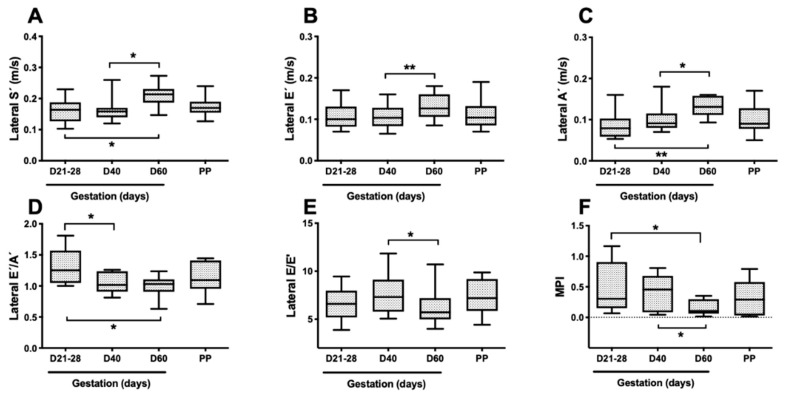
Box and whiskers plot showing LV systo-diastolic function assessed by TDI (**A**–**E**) and MPI (**F**). TDI parameters were obtained at the lateral margin of the mitral annulus. S’, peak systole in ejection phase; E’, peak myocardial velocity during early diastole; A’, peak myocardial velocity during atrial contraction; E, mitral peak flow velocity during early diastole. Thick line, box, and whiskers stand for median, interquartile range, 5th and 95th percentiles, respectively. * *p* < 0.05; ** *p* < 0.01.

Changes in TDI variables throughout pregnancy are shown in Figure 1. The myocardial velocities obtained at the septal wall (septal S’, E’ and A’) did not change throughout pregnancy. The E’ at the lateral margin of the mitral annulus increased significantly with advancing gestation, as values obtained at T3 were 24% higher than at T2 (*p* < 0.01). The lateral S’ and A’ were also higher at T3 than at T1 or T2 (*p* < 0.01). Lateral S’ was 35% and 30% higher at T3, and lateral A’ was 66% and 34% higher at T3 when compared to T1 and T2, respectively. The E’/A’ ratio decreased with gestational age, being 19% lower in T3 when compared to T1 (*p* < 0.05). The E/E’ ratio of the lateral mitral annulus decreased throughout pregnancy, being 6% lower at T3 compared to T1 (*p* < 0.01) and was always within normal limits as described for healthy dogs [11].

## 4. Discussion

Blood pressure values were elevated throughout the entire pregnancy (>160 mmHg), opposing a previous study carried out in small pregnant bitches, where systolic blood pressure did not change [8]; observed values were maximal at early pregnancy, as documented previously [5], and decreased until postpartum, where they returned to physiological values. The increase in blood flow resistance that occurs in early pregnancy in the bitch [28] might be the cause of the observed elevated blood pressures (SAP: 185.7 ± 19.37 mmHg vs. 130.9 ± 11.55 mmHg at postpartum). A possible explanation for the progressive decline of blood pressure during pregnancy relates to the decline in resistance index of the uterine artery by day 30 onwards [5], as a consequence of the development of a vascularized foetal chorioallantoic lamellae around the 25th day after conception [29]. With the development of the placenta-foetal circulation, the bitch blood flow resistance decreases, with concurrent decrease in arterial pressures.

Our data suggest that early systemic hypertension possibly triggers the following cardiac events: a pressure overload in canine early pregnancy and consequent increased left ventricular filling pressure (increased lateral E/E’) induces an increase in left atrium size by day 40. As the placental-foetal circulation develops [5], the decrease in peripheral vascular resistance increases cardiac output and heart rate (and a tendency to increase EDV and stroke volume by day 60), similarly to human early pregnancy. In this study, heart rate was maximal immediately prior to parturition, as previously described [4,5,19,30], suggesting a higher sympathetic tone. Interestingly, a recent study did not report an increase of heart rate during gestation [8].

In human gestation, it is well established that an early endocrine mediated fall in vascular tone triggers volume-restoring mechanisms, responsible for the increasing cardiac output seen through pregnancy, primarily caused by an increased stroke volume, but later on also due to increased heart rate [16]. This naturally occurring volume overload state induces a reversible LV eccentric hypertrophy [31], also observed in mice [32], and an increase in left atrium dimension that resolves postpartum [17]. Also, LV mass has been reported to increase, being associated with an elevated end-diastolic volume [18,33].

In the current study, no cardiac hypertrophy was observed, despite the tendency of some variables to increase at the end of gestation (e.g., LV mass, LV end-diastolic dimension). Previous studies reported contradictory results, with some reporting no increase of LV end-diastolic dimension or volume [4,8], whilst others reported an increase of these parameters during canine pregnancy [5,7]. Furthermore, increased thickening of cardiac walls (e.g., LVPWs) was observed in pregnant bitches, supporting the presence of cardiac hypertrophy, but only by the second half of the pregnancy, after the drop in systolic blood pressure [5,6]. In opposition to what is reported in pregnant women [17,34], we also failed to demonstrate atrial enlargement, in accordance to previous canine studies [4,5,8].

In bitches, several cardiac indices of systolic function increase in late pregnancy [4,5]. In this study, cardiac output was increased by 19.6% at day 60 of gestation, as compared to postpartum values, probably because of increased heart rate, as the stroke volume did not change throughout pregnancy. Despite the fact that FS and EF also did not change during gestation, Doppler-derived velocities of ventricular ejection reached their maximum values at late pregnancy, suggesting an improvement of systolic function. Concerning TDI parameters, we observed that S’ (lateral mitral annulus) significantly increased at late pregnancy, as recently reported [8]. These results suggest that the growing foetuses’ high demands are provided by an increase in both heart rate and contractility to achieve an optimal cardiac output. On the other hand, preload could affect TDI variables in clinical healthy dogs; specifically, ventricular overload is associated with increased S’, E’ and E’:A’ ratio [35], consistent with the results of the present study.

There are few studies that evaluated diastolic function in pregnant bitches. In human pregnancy, studies on mitral inflow velocities showed an increase in E wave on the first trimester of pregnancy [2,18], followed by a decrease with gestational age [3,9,10]. An increase in A wave through pregnancy, with a concurrent decreasing E/A ratio, was also observed in woman [2,3,9,10,18,36,37], and recently in bitches [8]. In our study, the peak transmitral inflow velocity during early diastole (E wave) was lower in early pregnancy, as compared to mid and late pregnancy, similar to a previous study [6], and postpartum period. Also, mitral valve A-wave maximum velocity significantly increased with pregnancy, in accordance to the same study [6] and the E/A ratio tended to reduce progressively as pregnancy advanced.

The compensatory increase in preload (increased plasma volume) observed in pregnancy [38] induces an increase in the LV filling rates (as evaluated by TDI E’ and A’) [9]. Myocardial hypertrophy, a possible consequence of this overload state, decreases LV compliance [18]; this eventually decreases early diastolic filling rate (E’), with the left atrium becoming responsible for the ventricle filling rate (increase A’) [9]. Contrary to previous studies in women [9], we observed an increase of the early diastolic filling rate (E’) in late pregnancy. We hypothesized that, although there is an increase in preload, the evaluated bitches did not reach the point of decreased left ventricular compliance (a significant hypertrophy throughout pregnancy was not detected). Additionally, as the left atrium assumes the main role in LV filling, the A’ wave increases with gestation and reaches a maximum value in late pregnancy (66% of early pregnancy), so E’/A’ratio and E/E’ratio decreased by 19% and 6%, respectively, from early to late pregnancy.

The MPI is a valuable tool in evaluating LV systo-diastolic function. MPI seems to be independent of ventricular geometry, preload and heart rate [14,15]. In the present study, MPI was higher at early pregnancy (0.47 ± 0.40), with values superior to those reported for healthy dogs of similar size (0.28 ± 0.12) [39], decreasing throughout pregnancy until achieving the lowest values at late pregnancy. These observations support an intrinsic contractility dysfunction associated with early pregnancy [39,40]. Similarly, other systolic function parameters (e.g., S’ of lateral mitral annulus) increased at late pregnancy, which is consistent with improvement of cardiac systolic function throughout gestation. On the contrary, studies performed in women demonstrated that MPI rises progressively during pregnancy and increased further after birth, suggesting a deterioration of cardiac function [36,41].

One potential limitation of the current study would be the sample size, since serial evaluation of cardiac function during pregnancy was often not feasible; however, we may assume that it is minimized as we have only included Saint Bernard bitches. As previously referred, we intended to characterize the cardiac adaptations during pregnancy in a large breed dog prone to develop cardiac diseases. However, the obtained results may not be representative of the large breed dog population.

## 5. Conclusions

The present work demonstrated an elevation of blood pressure throughout pregnancy, with the highest values observed in early pregnancy. Cardiac output increased during pregnancy, probably due to increased heart rate. Pregnancy, as a chronic, natural volume-overload state, also caused alterations on TDI parameters, where S’, E’ and A’ values increased with advanced gestation. On the other hand, the values obtained for MPI, in conjunction with other systolic function parameters, suggested an improvement of cardiac function throughout gestation in a large breed dog such as the Saint Bernard. In the future, increasing the sample size, or even including different breeds of the same size, would help to better describe these cardiac adaptations. It would also be interesting to study animals with different obstetric complications, to deepen the knowledge already published [6]; also, the study of pregnancy changes in animals with previous cardiac dysfunction would be relevant, although these bitches are not usually included in breeding programs.

## Figures and Tables

**Table 1:** Clinical and echocardiographic variables (mean ± SE). The P-value relates to the main effect of time on echocardiographic variables obtained at T1 (early pregnancy; days 21–28), T2 (mid pregnancy; day 40), T3 (late pregnancy; day 60), and PP (postpartum; 4 to 8 weeks after parturition). * *p*-value < 0.05, ** *p*-value < 0.01. BSA, body surface area. Rest of abbreviations as in the text.

Variable		T1 (21–28 d)	T2 (40 d)	T3 (60 d)	Postpartum (4–8 wk)	*p*-Value
BW (kg)		59.67 ± 5.93	65.13 ± 6.82	71.50 ± 6.82	61.03 ± 4.42	T3 vs. T1, T2 and PP **
BSA (m^2^)		1.53 ± 0.10	1.62 ± 0.11	1.72 ± 0.11	1.55 ± 0.08	T3 vs. T1, T2 and PP **
HR (bpm)		117.2 ± 11.16	120.4 ± 14.42	128.4 ± 7.88	118.6 ± 8.95	T3 vs. T1 and PP *
SAP (mmHg)		185.7 ± 19.37	167.6 ± 20.55	166.1 ± 25.47	130.9 ± 11.55	PP vs. T1, T2 and T3 **
MAP (mmHg)		139.8 ± 18.43	123.8 ± 19.29	118.9 ± 21.24	91.8 ± 9.73	PP vs. T1, T2 and T3 **
DAP (mmHg)		114.4 ± 18.2	100 ± 19.06	93.8 ± 18.55	70.5 ± 8.57	PP vs. T1, T2 and T3 **
LVDd (mm)		48.64 ± 4.32	48.14 ± 4.37	49.79 ± 4.56	47.82 ± 2.81	NS
LVDs (mm)		35.94 ± 4.94	36.63 ± 5.56	35.86 ± 4.33	34.49 ± 3.75	NS
LVPWd (mm)		11.37 ± 1.11	11.26 ± 0.88	11.46 ± 1.08	11.60 ± 1.31	NS
LVPWs (mm)		14.58 ± 1.56	14.27 ± 1.65	14.69 ± 1.58	15.47 ± 2.62	NS
IVSd (mm)		11.28 ± 1.87	10.79 ± 1.29	11.83 ± 2.22	11.28 ± 1.92	NS
IVSs (mm)		13.99 ± 2.32	13.57 ± 1.89	14.34 ± 2.37	13.81 ± 2.42	NS
LV mass (g)		207.89 ± 38.38	199.14 ± 51.17	227.39 ± 60.38	205.40 ± 40.15	NS
EPSS (mm)		7.08 ± 2.01	6.96 ± 2.37	5.87 ± 3.95	6.61 ± 0.81	NS
FS (%)		26.25 ± 5.88	24.22 ± 6.80	28.02 ± 4.99	27.97 ± 5.24	NS
EF (%)		55.87 ± 7.06	55.29 ± 9.03	58.63 ± 4.46	58.88 ± 4.35	NS
SV (mL)		56.37 ± 13.26	50.86 ± 9.43	63.32 ± 15.38	57.13 ± 10.43	NS
CO (L/min)		6.57 ± 1.49	6.12 ± 1.37	8.12 ± 1.96	6.79 ± 1.43	T3 vs. PP *
LA (mm)		38.85 ± 5.59	41.55 ± 9.25	38.90 ± 8.33	41.59 ± 7.19	NS
Ao (mm)		28.69 ± 2.80	28.80 ± 1.68	28.83 ± 2.40	29.25 ± 3.21	NS
LA/Ao		1.36 ± 0.16	1.44 ± 0.31	1.34 ± 0.24	1.43 ± 0.25	T3 vs. T2 *
AoV (m/s)		1.28 ± 0.19	1.28 ± 0.15	1.48 ± 0.16	1.32 ± 0.17	T3 vs. T1 **
E (m/s)		0.68 ± 0.08	0.75 ± 0.12	0.75 ± 0.13	0.76 ± 0.09	T1 vs. T2 and T3 *
A (m/s)		0.61 ± 0.07	0.71 ± 0.11	0.74 ± 0.15	0.72 ± 0.08	T1 vs. T2, T3 and PP *
E/A ratio		1.12 ± 0.08	1.04 ± 0.12	1.02 ± 0.12	1.07 ± 0.13	NS
Pul (m/s)		0.92 ± 0.16	0.95 ± 0.12	1.15 ± 0.16	1.05 ± 0.11	T3 vs. T1 and T2 *
MPI		0.47 ± 0.40	0.41±0.29	0.16 ± 0.12	0.33 ± 0.29	T3 vs. T1 and T2 *
Septal wall	S’ (m/s)	0.11 ± 0.02	0.12 ± 0.03	0.12 ± 0.02	0.13 ± 0.03	NS
	E’ (m/s)	0.09 ± 0.03	0.08 ± 0.02	0.09 ± 0.02	0.09 ± 0.02	NS
	A’ (m/s)	0.08 ± 0.03	0.08 ± 0.02	0.09 ± 0.01	0.08 ± 0.02	NS
	E/E’ ratio	8.44 ± 2.80	9.36 ± 2.35	8.95 ± 2.83	8.84 ± 1.95	NS
	E’/A’ ratio	1.21 ± 0.37	1.03 ± 0.18	0.97 ± 0.19	1.11 ± 0.20	NS
Lateral wall	S’ (m/s)	0.16 ± 0.04	0.16 ± 0.04	0.21 ± 0.04	0.17 ± 0.04	T3 vs. T1 and T2 *
	E’ (m/s)	0.11 ± 0.03	0.11 ± 0.03	0.13 ± 0.03	0.11 ± 0.04	T3 vs. T2 **
	A’ (m/s)	0.09 ± 0.03	0.10 ± 0.03	0.13 ± 0.02	0.10 ± 0.03	T3 vs. T1 and T2 **
	E/E’ ratio	6.68 ± 1.76	7.54 ± 2.16	6.15 ± 1.95	7.20 ± 1.83	T3 vs. T2 **
	E’/A’ ratio	1.30 ± 0.29	0.94 ± 0.32	1.00 ± 0.17	1.03 ± 0.14	T1 vs. T2 and T3 *

## Data Availability

All data analyzed during this study are included in this published article.
